# The Swedish military conscription register: opportunities for its use in medical research

**DOI:** 10.1007/s10654-022-00887-0

**Published:** 2022-07-09

**Authors:** Jonas F. Ludvigsson, Daniel Berglind, Kristina Sundquist, Johan Sundström, Per Tynelius, Martin Neovius

**Affiliations:** 1 Department of Medical Epidemiology and Biostatistics, Karolinska Institutet, Stockholm, Sweden; 2 Department of Paediatrics, Örebro University Hospital, Örebro, Sweden; 3 Division of Epidemiology and Public Health, School of Medicine, University of Nottingham, Nottingham, UK; 4 Department of Medicine, Columbia University College of Physicians and Surgeons, New York, NY USA; 5 Department of Global Public Health, Karolinska Institutet, Stockholm, Sweden; 6 Centre for Epidemiology and Community Medicine, Region Stockholm, Stockholm, Sweden; 7 Center for Primary Health Care Research, Department of Clinical Sciences Malmö, Lund University, Lund, Sweden; 8 Clinical Epidemiology Unit, Department of Medical Sciences, Uppsala University, Uppsala, Sweden; 9 George Institute for Global Health, University of New South Wales, Sydney, Australia; 10 Clinical Epidemiology Division, Department of Medicine Solna, Karolinska Institutet, SE-171 76 Stockholm, Sweden

**Keywords:** Conscription, Military, Register, Sweden

## Abstract

In Sweden, conscription around age 18y was mandatory for young men until June 30, 2010. From July 1, 2017, it became mandatory again for both sexes but the proportion of summoned people for standardised testing has so far been low. This paper describes the history, structure and content of the Swedish Military Conscription Register (SMCR). We retrieved information about the SMCR from written sources and through e-mail interviews with key personnel at the Swedish Defence Conscription and Assessment Agency. We also analysed data from the SMCR between 1969 and 2018. Between 1969 and 2018 the SMCR contains digital data on approximately 2 million individuals (98.6% men). Most conscripts were born between 1951 and 1988 (n = 1,900,000; tested between 1969 and 2006). For the 1951–1987 birth cohorts, the register has a population coverage of approximately 90% for men. Conscripts underwent written tests focusing on verbal, spatial, logical and technical ability, medical, physical, and psychological tests. The medical assessment included hearing, vision, muscle and exercise capacity, height, weight, blood pressure and resting heart rate. The SMCR has been widely used to study, e.g., obesity, cardiovascular disease, mental health, crime, cardiovascular fitness, muscle strength, sick leave and disability pension. Severe disease could qualify for exemption from military service. Thus, the prevalence of such diseases is underestimated in the SMCR population. Between 1990 and 2018, about 25,000 women also volunteered for testing. The SMCR contains population-based data on physical and psychological health in about 90% of all men born between 1951 and 1987 (corresponding to testing between 1969 and 2006), and can be used to address a host of research questions.

**Supplementary Information:**

The online version contains supplementary material available at 10.1007/s10654-022-00887-0.

## Introduction

In Sweden, conscription (defined as compulsory enrolment of persons for military service) was mandatory for young men until June 30, 2010. Almost all men were summoned for conscription at about 18y of age and a small fraction of women (about 25,000 women between 1990 and 2018), on a voluntary basis. For more than 40 calendar years, conscription data have been stored digitally in the Swedish Military Conscription Register (SMCR).

Conscription entailed medical, physiological and psychological testing. While the testing aimed to select young men suitable for military service and allocate them to different positions in the military, the register also offers unique opportunities for medical and social research as it contains population-based data on, for example, anthropometry, exercise capacity, muscle strength, blood pressure and cognition in virtually all 18-year-olds without chronic diseases independent of socioeconomic status.

One aim of this review is to present and explain the contents of the SMCR. A second aim is to present studies in which components of the register have been used and outline the potential future use of this register in medical research.

## History of the Swedish conscription

Compulsory military conscription for men aged 18–47y began in Sweden in 1901, following a decision in the Swedish parliament in 1873 that the national defence should be built on two pillars: permanently employed officers and male conscripts. Conscripts were typically enlisted for 7–15 months for initial training.

During the early 1900s, conscripts were subjected to extensive testing of muscle strength and cardiorespiratory exercise capacity while cognitive testing was introduced in the 1960s. Testing typically took place over 2 days. The purpose was to allocate men, according to their capabilities, to different positions in the Army, Navy and Air Force, or exempt them from the draft.

Between the world wars, the conscription system was reduced and 1/3 of all men were exempted. When tensions rose in Europe in the 1930s, the Swedish parliament again sought general conscription. After World War 2, with the cold war intensifying, a shift from limited to extensive conscription began in 1965, including physical examinations performed by physicians and interviews by psychologists. In 1968–1969, a new conscript registration system under The Swedish Conscription Authority was created and from 1969 digital conscription records are available. In the 1980s, women could join the military, but conscription remained mandatory only for men.

**Fig. 1:**
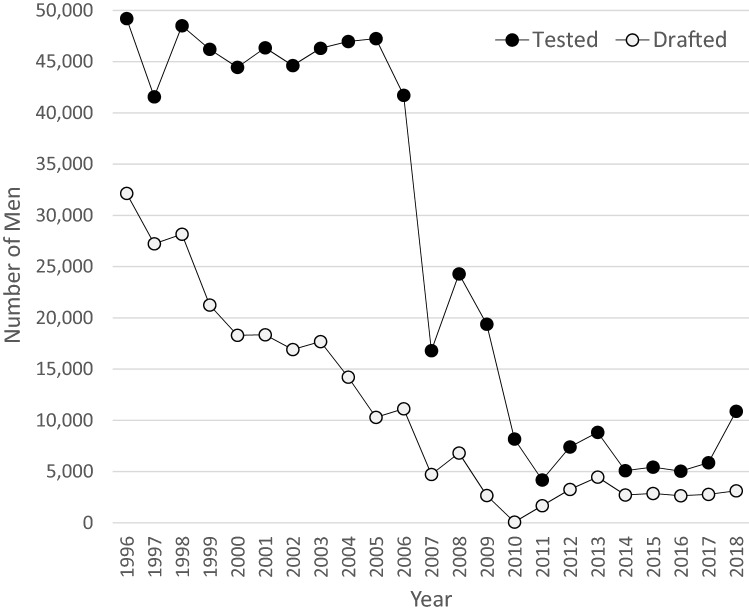
Number of tested per conscription test year (full black line) and number of drafted per year (dotted grey line) for the period 1996 to 2018

In the 1990s, the proportion of men undergoing conscription testing remained high (> 90% of the corresponding male birth cohorts), whereas the number of men drafted into military training decreased (Fig. 1), partly due to decreased tensions in Europe. In 1995, the Swedish Conscription Authority merged with the Civil Conscription Committee, forming the Swedish Defence Conscription and Assessment Agency.

In 2007, the Swedish Defence Conscription and Assessment Agency established an online form to pre-screen men potentially suitable for conscription (Tables 1 and 2). At this stage, the number of individuals assessed declined from more than 40,000 annually to about 20,000 (about 30% of the corresponding male birth cohorts) of whom only a small proportion was selected for service (Fig. 1).

**Table 1:** Interview questions before or during conscription: Health

Diseases frequently resulting in exemption from military service^a^ (Examples)
*Infectious disease*
HIV, active hepatitis
*Metabolic, cardiovascular, renal*
Thyroid disease (with symptoms despite treatment), any diabetes, BMI < 18^b^ or > 33
Renal disease, kidney failure, enuresis and encopresis^c^
*Neurological & psychiatric*
Multiple sclerosis, epilepsy and other neurological diseases, including cerebrovascular disease
Schizophrenia, delusions, bipolar disease, panic, anorexia, personality disorders and autism spectrum disease
Psychiatric diseases that could lead to exemption also include depression and anxiety if medical treatment, including therapy, had been needed in the past 2 years before conscription
*Vision, hearing, speech, balance*
Recurrent keratitis; iritis at least twice during the past 3 years. Retinal detachment or glaucoma
Strabismus with double vision, very poor vision (hyperopia > 8 or myopia < -9), lack of night vision, poor colour vision, visual field loss, one-eyed
Paradoxical vocal cord dysfunction
Otosclerosis, chronic or recurrent ataxia; poor balance
*Pulmonary*
Chronic pulmonary disease, recurrent pneumothorax
Asthma (after the age of 13y) with an impact on daily life
*Chronic inflammatory diseases*
Inflammatory bowel disease (IBD), celiac disease, cow’s milk allergy
Septic/Infectious arthritis, rheumatoid arthritis and severe joint disease
Inflammatory diseases such as systemtic lupus erythematosus (SLE), pelvospondylitis, inflammatory disease of the spine
Other skeletal disorders, including deformities of the spine or the pelvis
*Other*
Malignancies in the past 5 years
Haemolytic anaemia, coagulation disorders (including chronic immune thrombocytopenia purpura [ITP])
Allergy requiring adrenalin, steroid treatment or hospital care
Extensive eczema, psoriasis (extensive or affecting hands and feet), chronic or recurrent urticaria
Acne treated with isotretinoin in the past 6 months
*Additional health issues asked for in the pre-screening questionnaire from the military since 2007*^d^
Infectious disease, heart disease
Chronic prostatitis. Gynaecological problems
Raynaud’s disease
Headache, abdominal pain

^a^Diseases requiring (i) regular health care contact or (ii) treatment, or (iii) conditions that may deteriorate during military service. While these diseases generally led to exemption from military service, most of them (except those listed in the main text) have traditionally not been cause for exemption from conscription

^b^Not < 18.5 which is otherwise used by the World Health Organization to define underweight

^c^It is possible that also individuals with [certain forms of] pyelonephritis were exempted

^d^These health issues were included in the pre-screening questionnaires that the Swedish military began to distribute to potential conscripts in 2007, but exemptions for medical reasons were done also prior to 2007

**Table 2:** Interview questions before or during conscription: Education, personality and motivation

Other issues asked for during conscription/pre-screening (reflects questionnaire from 2021)^a^
Height and weight
Have you been frostbitten?
How often do you drink alcohol to get drunk?
Sleeping habits
Difficulties in controlling one’s temper; easily get into fights
Do you think it is important that Sweden has a military defence?
Have you used other drugs than alcohol?
Have you used anabolic steroids?
Frequency of physical exercise
Compare your physical fitness (and muscle strength) to that of your peers
Do you currently attend school? Did you achieve the necessary grades to start high school?
How do you grade your relationship with your teachers and classmates?
Did you ever consider dropping out of school?
Have you hit/beaten/knocked or harassed someone mentally on at least four separate occasions?
Questions about friends
Have you been convicted of a crime?
How do you manage stress?
Do you have or plan to acquire a driver’s license?
Are you interested in any of the following: sports, outdoor life, shipping, cooking, wildlife, computers, logical problem solving, driving vehicles, repairing vehicles, technology?

^a^Pre-screening questionnaires have been distributed to potential conscripts since 2007. Table 2 reflects the content of the pre-screening questionnaire for 2021

In 2010, a government decree stipulated that all individuals aged 16–18y must undergo conscription if the government decided, irrespective of sex. In practice, the conscription usually takes place around age 18y and from age 24y people do not undergo conscription unless special circumstances apply.

The universal conscription system was suspended on July 1, 2010, and replaced by permanent officers and voluntarily enlisted soldiers. The number of tested individuals decreased to a median 5000/year between 2011 and 2017 (< 10% of the corresponding male birth cohorts; Table 3; eFigure 1). On March 2, 2017, the Swedish government reactivated the conscription system, including both men and women, due to increased tensions in Europe and difficulty attracting recruits for the military forces. Only about 4000 are initially planned to be drafted annually, which is < 5% of each annual birth cohort of men and women, and then increase to about 8000 by 2025, according to the current plan. The number of tested men and women in 2018 was approximately 13,000 and 24% were women (Table 3; eFigure 1).

**Table 3:** Descriptive statistics for individuals tested between 1969 and 2018^a^

Test years	1969–1979	1980–1989	1990–1999	2000–2006	2007–2009	2010–2017	2018
n^b^	498 453	548 582	492 503	323 898	64 575	45 544	12 883
% Women	0%	0%	1%	2%	6%	18%	24%
% Nordic birth country	99.3%	98.1%	95.1%	92.1%	95.3%	93.3%	92.6%
*Age at testing (yrs)*							
Mean	18	18	18	18	18	20	19
Standard deviation	1	1	1	1	1	3	2
*Height (cm)*							
Mean	178.8	179.2	179.4	179.7	179.3	178.1^c^	177.5
Standard deviation	6.5	6.6	6.7	6.9	7.4	8.3	8.5
*Weight (kg)*							
Mean	68.2	70.0	71.8	73.9	73.9	74.9	74.1
Standard deviation	9.8	10.4	11.5	13.2	12.2	12.0	12.0
*Body mass index*							
Mean	21.3	21.8	22.3	22.9	23.0	23.6	23.5
Standard deviation	2.7	2.9	3.2	3.7	3.3	3.1	3.1
*Missingness*^*d*^	*2%*	*1%*	*5%*	*35%*	*30%*	*21%*	*36%*
*Estimated coverage*^*e*^							
Men	82%^f^	92%	94%	89%	32%	9%	21%
Women	0%	0%	1%	2%	2%	2%	7%

^a^For sex-specific data on anthropometry, please refer to eFigure 2 in the appendix

^b^Please refer to eFig. 1 in the appendix for number of tested for individual years

^c^The decreasing mean height from 2010 reflect the increasing share of women

^d^For height, weight and body mass index (BMI)

^e^Estimated by using birth cohort size as denominator

^f^Affected by that digital storage started in September 1969, missing conscription tests during the first 8 months of that year, and by the unexplained data loss in 1978

## Exemption from conscription and military service

The following individuals have been exempted from conscription and military service: (i) Swedish citizens living abroad, (ii) those having certain psychiatric disorders or special youth care, (iii) those receiving assistance allowance (or when parents receive care allowance for the youth) and (iv) those receiving support and service for certain functional impairments. The only religious basis for exemption is being a member of the Jehovah’s witness congregation.

Some diseases, or forms of these diseases, result in exemption from conscription, while a wider range of diseases usually lead to exemption from military service, but not necessarily from conscription (Table 1).

## Coverage

In total, the military authorities have digital conscription data on some 2 million individuals between 1969 and 2018 (Table 3, eFigure 1).

*Test years *1969–2006*:* Digital records of military conscription data are available from September 1969, including data from about 40,000–60,000 men per year until 2006, corresponding to about 90% of birth cohorts in 1951–1988 (Table 3). Data from the test year 1978 are only available for about 15,000 conscripts. This low rate is due to unexplained data loss, rather than much fewer individuals being tested. This data loss means that < 20% of the male birth cohort of 1960 (about 7000 of them tested in 1978) cohort have data in the register.

*Test years 2007–2009:* In 2007–2009 the number of tested dropped to 17,000–25,000 per year (Table 3; Fig. 1), reflecting substantial selection.

*Later test years:* From July 1, 2010, to March 1, 2017, mandatory military conscription was suspended and the median number of tested between 2010 and 2017 was around 5000 individuals (Fig. 1), reflecting even greater selection. In 2018, the conscription of 18-year-olds was re-instated and almost 13,000 individuals were tested (24% women).

## Description of conscripts

Conscription in Sweden was performed in a standardised fashion. The mean/median age at conscription between 1969 and 2006, i.e., when the population coverage was high, was 18.3/18.2y (10th percentile 17.8, 90th percentile 18.9y). Some 2% were > 22y old at conscription [1].

Tests were generally rated on a 9-point stanine scale (short for STAndard-NINE scale) that approximates a normal distribution with mean 5 and standard deviation 2. Higher values indicate a better outcome. Four percent of the conscripts scored an overall 9/9.

## Background data

*Conscript ID:* Between 1902 and 1953, conscripts and their results were tracked through unique military enrolment numbers. In 1953, enrolment numbers were replaced by personal identity numbers (PINs)[2] automatically containing data on age and sex (although women made up a minute proportion).

*Pre-testing questionnaire:* Since 2007, potential conscripts completed an extensive pre-testing questionnaire (Tables 1 and 2). This questionnaire was slightly modified over the years. In addition to the content listed in Tables 1 and 2, it also contained questions of earlier voluntary military service (the local home guards), as well as language skills other than Swedish, English, German, French or Spanish.

*Smoking and snuff.* Self-reported smoking has been recorded and categorised into seven categories (0, 1–10, 11–20, > 20 cigarettes/day and 1, 1–2, > 2 packages of tobacco/week)[3]. Please note that “0” is relevant for both cigarettes and packages of tobacco. For packages of tobacco, the two first categories overlap.

## On-site testing

In summary, the conscripts underwent several written tests focusing on verbal, spatial, logical and technical ability, (ii) a telegraph test, (iii) certain medical and physical tests and (iv) psychological evaluation. Only conscripts with a result above a certain threshold on the cognitive test, undertook the telegraph test.

Conscripts also had their hearing, vision, height, weight, blood pressure, resting heart rate, strength and exercise capacity tested or measured. Based on the results, a test officiator performed a short discussion with each conscript to decide whether the conscript should be exempted from military service, or more commonly, to what position and location the soldier should be assigned.

Only *licensed* nurses and psychologists were engaged in the testing. Psychologists also received additional training within the military organisation, and up until 1989, there were regular exercises to increase consistency between psychologists. Physicians involved in conscription testing had to be consultants with at least a few years of clinical training after specialisation.

## Anthropometry

At conscription, the participants had their height and weight measured and body mass index (BMI) calculated. The development of height, weight and BMI between 1969 and 2018 in tested individuals are shown overall in Table 3 and by sex in eFigure 2. While the pre-screening questionnaire introduced in 2007 also requested self-reported height and weight, anthropometric data from the cohorts most often used in medical research (conscription until 2006) were derived from medical examination.

## Physical capacity

*Ergometer test:* Conscripts had their cardiorespiratory exercise capacity examined using an ergometer bicycle (results were recorded in watts (W)). Only conscripts without disease or injury and normal electrocardiography were allowed to perform the bicycle test. After 5 min of bicycling (pulse between 120 and 170 beats/min), resistance was increased from a low level (initial resistance determined by weight (e.g., 125 W for weight 70 kg) by 25 W per minute until the conscript interrupted due to exhaustion [4].

Bicycling was performed at 60–70 revolutions per minute. If a conscript did not attain 180 heartbeats per minute, the test officiator could choose to re-test the conscript. There is a strong correlation (correlation coefficient r = 0.88) between the described maximal power output test (Wmax) and maximal oxygen uptake (VO_2_max) [5]. Moreover, VO_2_max can be calculated using the validated Eq. 1.76× (watts × 6.12/body weight (kg)] + 3.5. Thus, a Wmax of 270 W for a young adult weighing 70 kg translates into ≈42 mL/min × kg [6]. A change in examination protocol was probably established in August 1984 as a minor shift in exercise capacity was noted at that time (likely a more frequent stepwise load was used during testing [4, 7].

*Blood pressure:* A written protocol was used for blood pressure measurements. First, the conscript had his systolic and diastolic blood pressures measured after 5–10 min of rest in the supine position using an appropriately sized cuff at heart level. By tradition, Swedish physicians regarded supine blood pressure measurement as the gold standard. One measurement was made if the systolic blood pressure was ≤ 145 mm Hg and the diastolic pressure 50–85 mm Hg, but values outside these ranges prompted another measurement. These second values, even if outside the normal range, were then recorded. Test officiators were instructed to round the blood pressures to the nearest even number, but rounding to the nearest 5 and 10 mm Hg also occurred [8].

*Resting heart rate:* With the conscript in the supine position, resting heart rate was recorded in conjunction with the blood pressure measurements.

*Electro-cardiogram:* Because military service is characterised by frequent physical exertion, conscripts underwent ECG to identify disease (such as hypertrophic/dilated cardiomyopathy, myocarditis and arrhythmias) that could predispose to sudden cardiac death [9]. Abnormal ECG findings have been seen in about 10% of conscripts in Sweden [10].

*Muscle strength*: Until 1993/94, muscle strength was assessed by three tests: knee extension strength, handgrip strength and elbow flexion strength [4]. Each muscular strength component was measured using a validated isometric dynamometer test (50–999 Newton). All testing equipment was calibrated daily. A weighted sum of maximal knee extension (weighted × 1.3), elbow flexion (weighted × 0.8) and handgrip (weighted × 1.7), each measured in Newton with a dynamometer, has previously been used as a proxy for total body strength [11]. The Swedish Defence Conscription and Assessment Agency regarded the exact measurement protocol as confidential and has not revealed its content. However, researchers have observed (in preliminary analyses) that no systematic differences existed in the mean values of the measures between conscription offices, suggesting that a uniform protocol was used.

From 1994 and onwards, a single strength test was used called the IsoKai, which is a device used for measuring isokinetic muscular performance during a vertical lifting procedure [12, 13]. Under standardised conditions, the conscript was asked to counter a certain hydraulic pressure. The ISOKAI isokinetic lift test has been described as a highly reliable [12] and valid [13] (as compared to deadlift) test for maximal dynamic muscular strength.

## Cognitive ability

Cognitive testing started in the 1940s, but over time, the testing regimen changed. For some time, four domains were covered: synonyms, induction, spatial capacity, and technical understanding (1). Each domain contained 40 questions [1]. Carlstedt and Mårdberg [14] performed a thorough review of cognitive testing in the 1990s.

In 1994, the CAT-SEB (Cognitive Analytic Therapy-Swedish Enlistment Battery) test was introduced to evaluate the general intelligence of conscripts, including tests of verbal, spatial, logic inductive and technical ability (10 sub-tests) [15]. The four scores were converted to a stanine scale (a 9-point scale with a normal Gaussian distribution) by the conscription authorities and combined into a single G-factor, also on a stanine scale. For the IQ test, stanine 5 represents an IQ of 100. The IQ test does not discriminate against individuals with dyslexia. This conscription IQ test is similar to the Wechsler Adult Intelligence Scale.

It was not possible to avoid conscription by having low cognitive scores. The scores were only used for the precise military service assigned among those with a higher IQ.

## Psychological evaluation

Each conscript was interviewed for 20–30 min by a psychologist using a questionnaire and a semi-structured interview form to assess the conscript’s stress resilience (during military service or actual war), leadership skills and suitability for military service [1, 16]. Details of these interviews and the psychological testing have been reviewed by Lindqvist and Vestman based on an interview in 2004 with Johan Lothigius, chief psychologist at the Swedish Defence Conscription and Assessment Agency [1].

During the interview, the psychologist had access to other test results and answers from questions exploring the conscript’s friends, family, hobbies and school grades. One reason for conducting an interview, rather than just relying on an interview form, was to identify conscripts with anti-social disorders. Psychological profiling was initiated in 1969 and did not change until 1995, when minor refinements were made to the test.

Traits reflecting a high overall grade at the psychological evaluation were independence, outgoing character, persistence, emotional stability, power of initiative and willingness to assume responsibility [1]. Importantly, perceived motivation to do military service was not part of the grading process. The psychologist also evaluated to what extent the conscript could adjust to military life (restricting personal freedom). Among the variables recorded were claustrophobia and fear of heights.

Psychologists also assessed leadership qualities in conscripts. Only men with a stanine score ≥ 6 could become sergeants and those with ≥ 7 s lieutenants.

Another aim of the psychological testing was to identify people unfit for military service (e.g., expressing fundamental undemocratic values) [1]. Also, conscripts with an obsession with the military, or men with signs of anti-social personality disorders, were generally deemed unsuitable for military service (Table 2) [1].

## Other data

*Visual acuity.* Visual acuity was tested using Snellen charts (highest stanine test score is 9) [17].

*Hearing*. Hearing ability is divided into three levels: A (best, hearing < 25 dB), B (good) and C (poor). Hearing level A is required for certain military positions (for instance, naval sonar operator). The military may also choose not to place a conscript with poor hearing in a certain position where heavy sound exposure is frequent (protective measure).

*Biological data.* Finally, the SMCR contains limited data on erythrocyte sedimentation rate (ESR), erythrocyte volume fraction and proteinuria [18]. Data on proteinuria were obtained through a dipstick (categories: negative, trace, positive).

SMCR variable content varies over time and researchers should contact the Swedish Defence Conscription and Assessment Agency for a list of variables pertaining to their specific interest and time period.

## Ethics and how to access additional medical data

Approval from the Swedish Ethics Review Authority is needed to obtain data from the SMCR. Data can then be requested from the Swedish Defence Conscription and Assessment Agency and the National Military Archive. These agencies will first assess the researcher’s need for the requested data. As in most large-scale register-based studies in the Nordic setting, the need for individual consent is generally waived and de-identified data are delivered to the research group requesting data [19].

Using the personal identity number of each individual who have undergone military conscription testing, the information in the SMCR can be linked with other nationwide registers to collect both follow-up and medical history data, as well as socioeconomic and demographic data, from the National Board of Health and Welfare and Statistics Sweden [2, 20–22]. Linkage to the Swedish Total Population Register, with information on migration and date of death, allows for a virtually complete follow-up of individuals.

## Discussion

The SMCR contains digital data on approximately 2 million individuals between 1969 and 2018, with coverage of about 90% for men born between 1951 and 1988 (and conscription 1969–2006). Data from this register have been used as exposures and outcomes, as well as covariates to reduce potential confounding, and describe study populations.

The register has undergone major changes over time. A change in the distribution of exercise capacity was noted in August 1984 (likely due to modifying the examination protocol) [4], which should be considered when evaluating exercise capacity before and after 1984. The proportion of young men undergoing conscription decreased dramatically in 2007, and the test year 1978 has a lot of missing data, probably for administrative reasons. Moreover, there are some minor changes in the MSCR, overall and for specific variables. Resting heart rate measurements are almost completely missing for 1984–1993. A valid resting heart rate measurement was available for about 1 million men, a valid systolic and/or diastolic blood pressure measurement for 1.6 million men [23].

The proportion of men tested fell markedly in 2007, which diminishes the register’s statistical power and representativity from 2007 and onwards (eFigure 1).

Conscription data have been used to assess a wide range of measures. Common to many medical studies is that they use data on exposures from young adulthood for longitudinal studies of outcomes later in life, sometimes extending to old age. In a case–control study Sundin et al. found that 1.8% of conscripts with future end-stage renal disease had an ESR ≥ 15 mm/h at conscription compared to 0.4% in controls. A high ESR may on the other hand be inversely related to later Parkinson’s disease [24]. Another study found that low-stress resilience (a score of 1–3 out of 9) in Swedish men born 1952–1956 (conscription about 1970–74) was linked to an increased risk of stroke in adulthood [25].

The register has also been used for outcome assessments [17, 26], especially to study the role of perinatal factors and markers of health in young adulthood [27–29].

Members of our research group and others have previously studied the prevalence of obesity [30–32], and its association with mortality [33, 34] and cardiovascular health [8, 35–39]. Still other researchers have demonstrated that heart rate and blood pressure at conscription may be linked to the risk of psychiatric disorders later in life [23, 40]. In young men, smoking and excessive alcohol intake have been linked to a moderately increased risk of later depression [41]. Other studies have used the register to investigate sick leave and disability pension [42–45].

Studies have also explored health outcome in young men according to their overall muscle strength, grip strength and fitness [4, 46, 47]. Hyioshi et al. demonstrated that men with poor vision had a higher rate of cycling injuries [48]. The association between military deployment abroad and mental health, mortality, violent crime, marriage and divorce has also been investigated [49–52]. The assessment of conscripts by a psychologist has been linked to labour market outcomes (e.g., earnings and unemployment) [1] and associated with managerial vs. non-managerial positions [1].

## Comparison with other Nordic conscription databases

Conscription registers are available in several Nordic countries. In *Norway*, all Norwegian citizens (only males up until 2007) had to undergo a health board examination for conscription [53]. Norwegian data revealed that 73% of eligible men born in 1950, but 95% of eligible men born in 1960–91 were included in the Norwegian Armed Forces Health Registry [53]. This percentage compares to approximately 90% of men born in 1951–1988 in Sweden. Most young men in Norway undergo conscription just after 18y of age.

Norwegian conscription consisted of physical and cognitive tests, as well as a clinical examination. In Norway, all Norwegian citizens had to undergo a health board examination for conscription [53]. Medical conditions of the conscripts have been recorded using international classification of disease codes since the early 1970s.

Similar to the SMCR, the *Danish* correlate consists almost exclusively of men (Table 1 in their paper, Danish Conscription Database) [54]. In addition, conscription in Denmark is compulsory, except for young men with a history of severe disease, such as epilepsy and diabetes [54]. The coverage in the Danish register is estimated at 92% (of men born 1959–1984). After that period, similar data have been collected in the National Archives Database (1987–2011: n = 658,000, the Danish Defence Personnel Organisation Database (1995–2005: n = 219,000) and the Danish Conscription Register (2006–2015: n = 364,000)[54]. Variables recorded are similar to those in a Swedish setting. Female volunteers have been accepted in the Danish military since 1962, and since 2006, all women in Denmark are invited for conscription after their 18th birthday.

## Strengths and limitations

One strength of the SMCR is the standard data collection practices. Such standardisation increases the validity of the data collected. Along with the many years the register has been operative, researchers can access high-quality data for almost all men born in Sweden in the 1950s, 1960s, 1970s and part of the 1980s. Data from these birth cohorts are likely to represent the average male because conscription was mandatory and exemptions were few. Through the PIN [2], data can be linked to other nationwide population and healthcare registers. Finally, the large number of conscripts in the SMCR allows for the study also of rare events.

One limitation is the exclusion of individuals with certain pre-existing conditions. This means that the prevalence of certain diseases is underestimated in cohorts based on the SMCR. Despite this limitation, the data source is still likely to be useful for etiological research [55].

In conclusion, the SMCR is a comprehensive tool for medical researchers eager to examine the relationship between health in young adulthood, and earlier risk factors as well as future health and social trajectories, particularly in men**.**

## Supplementary Information

Below is the link to the electronic supplementary material.

Supplementary file1 (DOCX 325 kb)

## Abbreviations

BMI: Body mass index
ESR: Erythrocyte Sedimentation Rate
PIN: Personal identity number
SMCR: Swedish Military Conscription Register
