# Supplementary material for: The Swedish military conscription register: opportunities for its use in medical research
Source: Eur J Epidemiol. 2022 Jul 9;37(7):767–77. doi: 10.1007/s10654-022-00887-0 (PMC9329412; doi:10.1007/s10654-022-00887-0)
Supplement: Supplementary file 1 — Supplementary file1 (DOCX 325 kb) [file 10654_2022_887_MOESM1_ESM.docx]

**SUPPLEMENTARY APPENDIX**

**The Swedish Military Conscription Register: Opportunities for Its Use in Medical Research**

Jonas F. Ludvigsson, Daniel Berglind, Kristina Sundquist, Johan Sundström, Per Tynelius, Martin Neovius

### Table of Contents

| **Item** | **Description** | **Page** |
| --- | --- | --- |
| eMethods | History of the Swedish conscription | 2 |
| eFigure 1 | Number of tested and sex distribution during the period 1969 to 2018. | 4 |
| eFigure 2 | Height, weight and BMI in Swedish military conscripts from 1969 to 2018. | 5 |

**History of the Swedish conscription**

Conscription in Sweden began in 1901. This recruitment process followed a decision in the Swedish parliament in 1873 that the Swedish defence should be built on two pillars: permanently employed officers and male conscripts from the general population. Military service was compulsory for men aged 18 to 47y and substantially prolonged compared to the 1800s when conscript training usually took place during the summer months only. Barracks were built (during summers, only canvas tents had been needed), and conscripts were trained throughout the year. Typically, conscripts were enlisted for 7 to 15 months for initial training, although some enlistment periods were longer in the Navy.

During the early 1900s, conscripts were subjected to extensive testing of muscle strength and cardiorespiratory exercise capacity while cognitive testing was introduced in the 1960s. Testing typically took place over 2 days. The purpose was to allocate men, according to their capabilities, to different positions in the Army, Navy and Air Force, or exempt them from the draft.

Between World War 1 and World War 2, the conscription system was reduced and a third of all men were exempted from conscription. When tensions rose in Europe in the 1930s, the Swedish parliament again sought general conscription in the late 1930s and early 1940s.

After World War 2, Swedish politicians expected an international détente and general disarmament. However, with the cold war intensifying worldwide, a shift from limited to extensive conscription began in 1965, including physical examinations performed by physicians and interviews by psychologists. In 1968-1969, a new conscript registration system under The Swedish Conscription Authority was created with regional conscript registration offices. In the 1980s, women could join the military, but conscription remained mandatory only for men.

In modern times Swedish conscription has been regulated by four laws:

1. Military conscription in the future (Law: SOU 1984:71)
2. Military conscription in society (Law: SOU 1985:36)
3. Unarmed conscription in the future (Law: SOU 1986:30)
4. Exemption from military service (Law: SOU 1986:43).

In 1994, the law on total defence duty (Law1994:1809) was initiated. It applies to all Swedish residents (citizens and non-citizens) aged 16-70y.

In the 1990s, the proportion of all men undergoing conscription testing remained high, whereas the number of men drafted into military training decreased (**eFigure 1**). This difference was partly due to decreased tensions in Europe. In 1995, the Swedish Conscription Authority merged with the Civil Conscription Committee, forming the Swedish Defence Conscription and Assessment Agency. By then, the number of enrolment offices decreased to only six. Today, there are currently two enrolment offices (in Malmö and Stockholm), with a third site scheduled for 2022 (Gothenburg). The Swedish Defence Conscription and Assessment Agency is otherwise situated in Karlstad.

In 2007, the Swedish Defence Conscription and Assessment Agency established an online form to pre-screen men potentially suitable for conscription (**Tables 1** and **2**). At this stage, the number of individuals assessed declined from more than 40,000 annually to about 20,000, of whom only a small proportion was selected for service (**eFigure 1**).

In 2010, a government decree (law: 2010:447) stipulated that all individuals aged 16-18y must undergo conscription if the government decided, irrespective of sex. In practice, the conscription takes place during the 18^th^ year of life and generally no later than 19y of age. Individuals aged 24y or more are not obliged to undergo conscription if special circumstances do not apply (such a circumstance may be that a person has purposely absconded conscription). Foreign-born nationals becoming Swedish citizens after the age of 24y can still apply for military service.

The universal conscription system was suspended on July 1, 2010 and replaced by officers (permanent) and voluntarily enlisted soldiers. The number of tested individuals decreased markedly to a median 5000 annually between 2011 and 2017 (**eFigure 1**). In peacetime, the permanent officers were responsible for training enlisted soldiers. In wartime they were expected to serve as commanders. Due to the limited size of the peacetime organisation, voluntarily enlisted soldiers also assumed commander roles, but only at the lower levels (corporals, sergeants and second lieutenants) (Law: SOU 1984:71).

On March 2, 2017, the Swedish government reactivated the conscription system. Two reasons have generally been advanced for the reactivation: i) increased tensions in Europe and ii) difficulty attracting recruits for the military forces. A major change in 2017 was that the reactivated conscription system included both men and women. The first age cohorts requested by law to undergo conscription in 2017 were those born in 1999-2000. Only 4000 draft prospects per year are planned to be drafted compared to a size per birth year cohort of about 100,000 men and women. The number of tested men and women was approximately 13,000 in 2018 and 24% were women (**eFigure 1**).

**Two types of military organisation**

Between 1901 and 2010, Sweden had two types of military organisation. A smaller one, the Peacetime Organisation, for training purposes including training of conscripts, and a larger one, the War Organisation, at times of war.

**Exemption from conscription**

The following individuals have been exempted from conscription and military service: i) Swedish citizens living abroad, ii) those having certain psychiatric disorders or special youth care, iii) those receiving assistance allowance (or that parents receive care allowance for the individual) and iv) those receiving support and service for certain functional impairments. The only religious basis for exemption is being a member of the Jehovah’s witness congregation.

Also, individuals with any of the following *diseases, or certain forms of these diseases,* are frequently exempted from the draft: diabetes, inflammatory bowel disease (IBD), rheumatic disease, multiple sclerosis (MS), epilepsy and some other specified neurological disorders, heart disease, coagulation disorders, contagious diseases (e.g., HIV and hepatitis), severe allergy that may need emergency treatment, neuropsychiatric disease, psychiatric disorders and disability after an earlier accident. A wide range of other diseases usually led to exemption from *military service*, but not necessarily from conscription (**Table 1**).


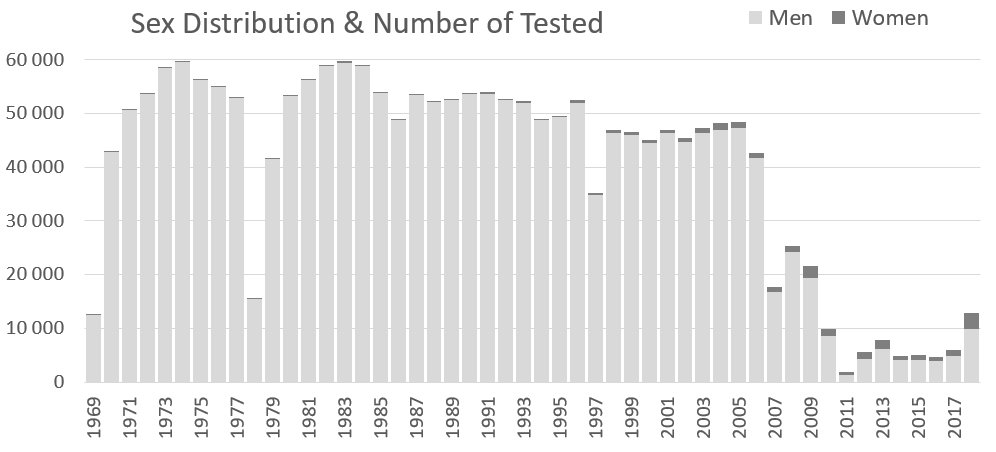


**eFigure 1.** Number of tested and sex distribution during the period 1969 to 2018.


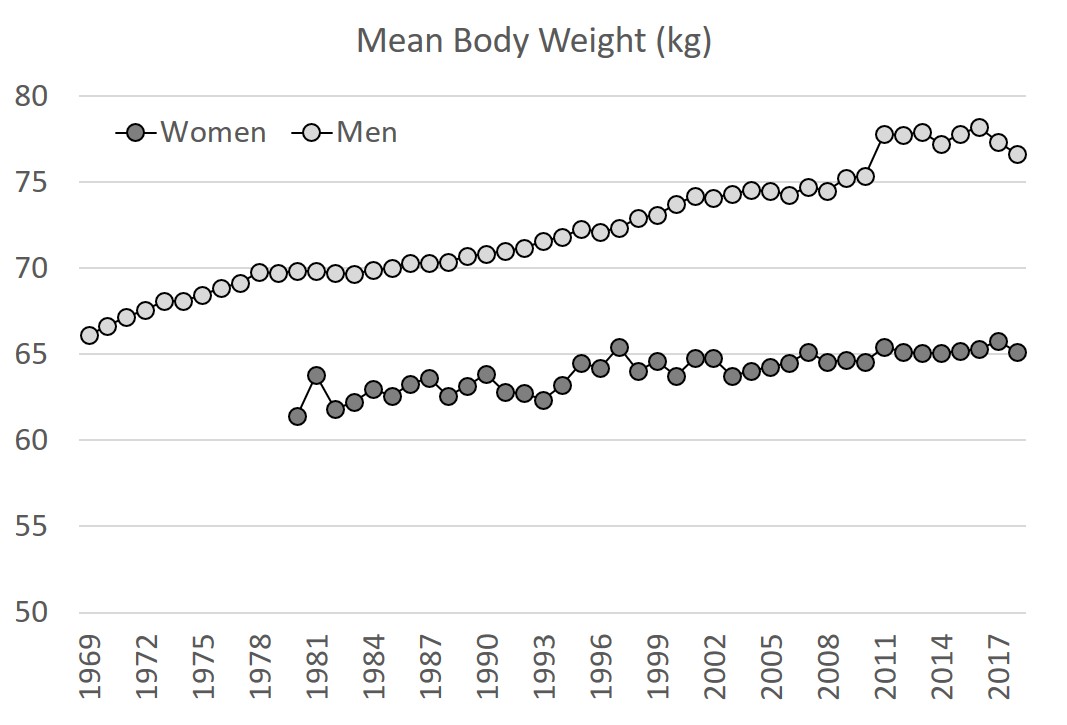


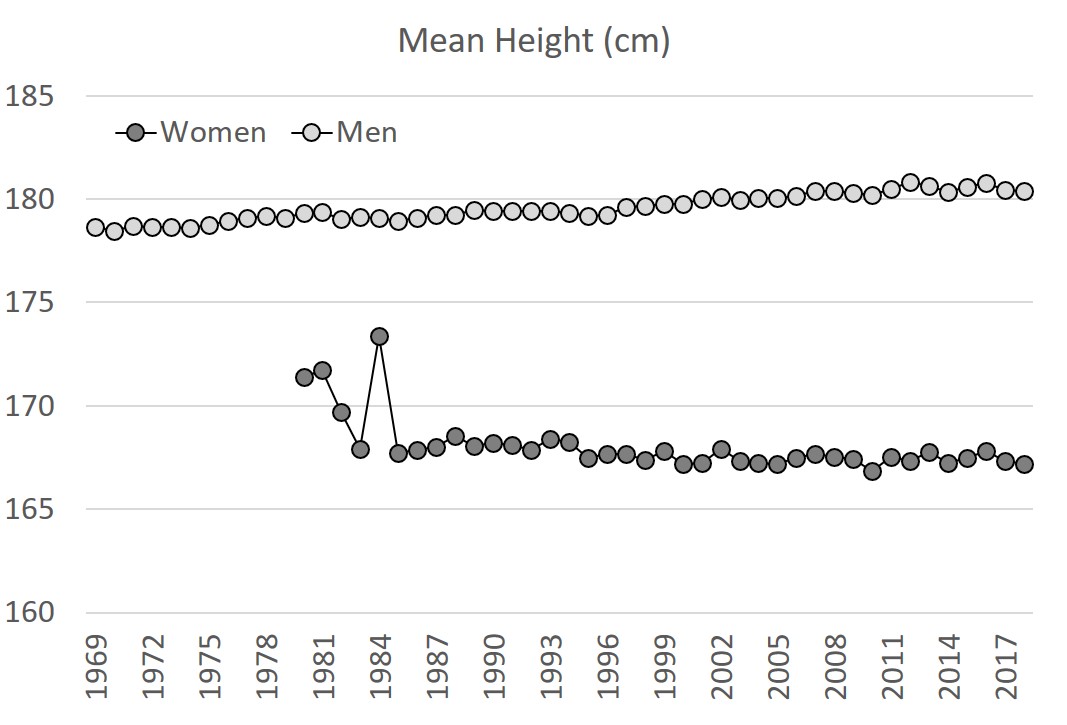


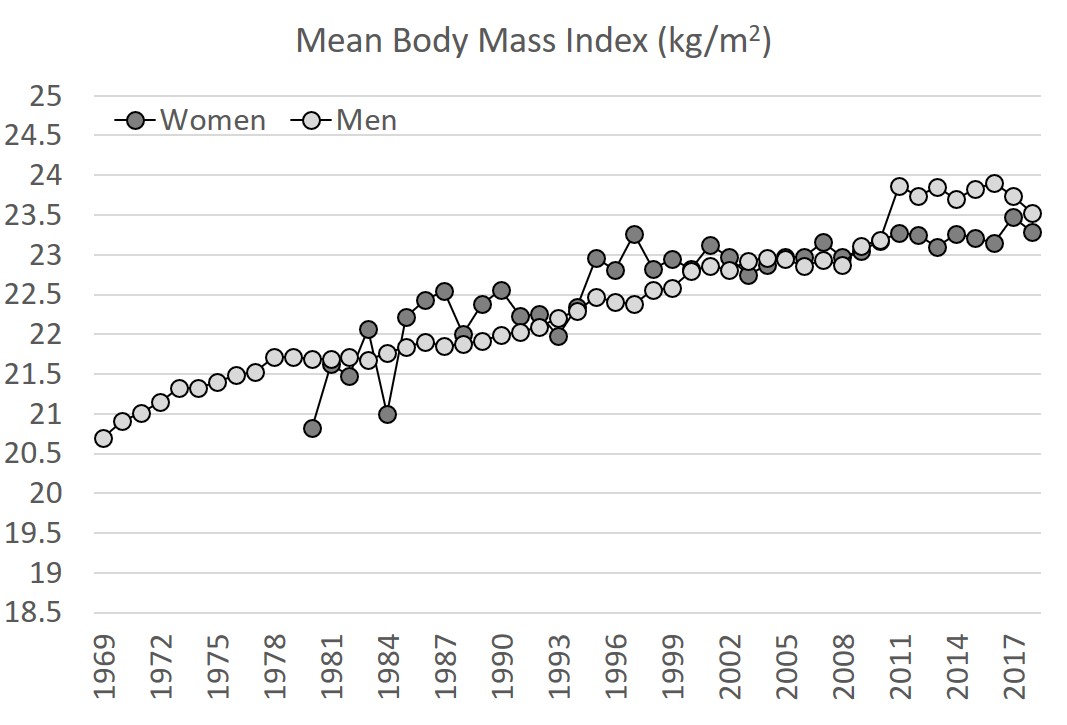


**eFigure 2.** Height, weight and BMI in Swedish military conscripts from 1969 to 2018.
